# Management Strategies for Aggressive Cushing's Syndrome: From Macroadenomas to Ectopics

**DOI:** 10.1155/2012/685213

**Published:** 2012-08-09

**Authors:** Carlotta Pozza, Chiara Graziadio, Elisa Giannetta, Andrea Lenzi, Andrea M. Isidori

**Affiliations:** Pathophysiology Section, Department of Experimental Medicine, Sapienza University of Rome, Viale del Policlinico, 155-00161 Rome, Italy

## Abstract

Cushing's syndrome (CS) is a rare but severe clinical condition represented by an excessive endogenous cortisol secretion and hence excess circulating free cortisol, characterized by loss of the normal feedback regulation and circadian rhythm of the hypothalamic-pituitary axis due to inappropriate secretion of ACTH from a pituitary tumor (Cushing's disease, CD) or an ectopic source (ectopic ACTH secretion, EAS). The remaining causes (20%) are ACTH independent. As soon as the diagnosis is established, the therapeutic goal is the removal of the tumor. Whenever surgery is not curative, management of patients with CS requires a major effort to control hypercortisolemia and associated symptoms. A multidisciplinary approach that includes endocrinologists, neurosurgeons, oncologists, and radiotherapists should be adopted. This paper will focus on traditional and novel medical therapy for aggressive ACTH-dependent CS. Several drugs are able to reduce cortisol levels. Their mechanism of action involves blocking adrenal steroidogenesis (ketoconazole, metyrapone, aminoglutethimide, mitotane, etomidate) or inhibiting the peripheral action of cortisol through blocking its receptors (mifepristone “RU-486”). Other drugs include centrally acting agents (dopamine agonists, somatostatin receptor agonists, retinoic acid, peroxisome proliferator-activated receptor **γ** “PPAR-**γ**” ligands) and novel chemotherapeutic agents (temozolomide and tyrosine kinase inhibitors) which have a significant activity against aggressive pituitary or ectopic tumors.

## 1. Introduction

Cushing's syndrome (CS) is a rare but severe clinical condition caused by cortisol excess of various etiologies. It is associated with significant morbidity and mortality and leads to metabolic, cardiovascular, infectious, psychiatric, and gonadal complications (Table 1). This complex endocrine disorder is a challenge in terms of efficient treatment. This paper will focus on traditional and novel medical therapy for hypercortisolism secondary to ACTH-secreting pituitary macroadenoma or carcinoma (Cushing's disease, CD) or to ectopic ACTH secretion. 

The natural history of *pituitary adenomas* varies widely. In the majority of cases, ACTH-secreting pituitary adenomas are small (<1 cm in diameter) and confined within the sella turcica. Pituitary microadenomas have a typically indolent growth rate, and clinically significant invasion and malignant transformation remain uncommon. However, 4–10% of patients present with larger tumors (>1 cm in diameter). These can cause symptoms due to mass effect before any full endocrine manifestations. Moreover, they are more refractory to surgical treatment and show a more unfavorable prognosis than microadenomas. For their behavior, presentation, and outcome, ACTH secreting macroadenomas present a distinct profile compared with microadenomas, although they probably represent one end of a spectrum of tumor autonomy, with specific growth and biochemical characteristics [1]. Morbidity and mortality are high with aggressive tumor behavior [2]. The 2004 WHO classification of pituitary adenomas now includes an “atypical” variant, defined as an MIB-1 proliferative index greater than 3%, excessive p53 immunoreactivity and increased mitotic activity. In the absence of metastases, however, invasive or aggressive pituitary tumors are not considered malignant. Pituitary carcinomas, defined as primary tumors with intra- or extracranial metastases, are rare, encountered in less than 1% of all hypophyseal tumors. They generally secrete ACTH or Prolactin. 

*Ectopic ACTH Secretion (EAS)* accounts for 15–20% of cases of Cushing's syndrome and covers a spectrum of tumors from undetectable isolated lesions to extensive metastatic and aggressive malignancies. EAS is often associated with severe hypercortisolemia causing hypokalemia, diabetes, generalized infections, hypertension, and psychotic reactions. Isidori et al. [3] proposed a classification based on the detection of the source of ectopic secretion. EAS is defined as *overt* when the tumor source is easily detected during the initial endocrine and radiological investigations, *covert *in patients presenting with hypercortisolemia where the ectopic source is not detected during initial tests but is discovered on subsequent evaluation or during prolonged followup, and *occult* when the patient's clinical features suggest CS and all tests indicate an ectopic source, but the primary lesion is not identified even after prolonged and repeated followup. Occult EAS is one of the most intriguing challenges for the clinical endocrinologist, as in some cases no tumor is found even after long-term followup or on autopsy [3]. The overall prognosis of patients with ectopic ACTH secretion is primarily determined by the nature of the underlying malignancy and the tumor stage on diagnosis.

## 2. Management of Cushing's Syndrome

Management of patients with CS requires a major effort to understand the etiology and to control hypercortisolemia as soon as the diagnosis is established. The most appropriate management of ACTH-dependent CS derives from a multidisciplinary approach that includes endocrinologists, neurosurgeons, oncologists, and radiotherapists.

The definitive treatment of CS consists in surgical resection of the tumor secreting ACTH. When the source of the excessive secretion *is the pituitary gland,* the standard approach is to perform an endoscopic endonasal trans-sphenoidal exploration, with excision of the tumor, if found. This surgical procedure is demanding and should only be performed in centers with extensive experience, to minimize operative risks, reduce the possibility of remission, and maintain other pituitary functions. It is successful in about 70% of cases (defined by suppressed plasma cortisol levels and normal 24 h urinary free cortisol) [4]. Success rates can reach 90% in selective adenectomy of microadenomas (<10 mm in diameter), but decrease to 65% for macroadenomas [5]. About 20% of tumors recur, and recurrence is more likely (and quicker) in larger than in smaller tumors. 

Pituitary irradiation achieves eucortisolism in 50–60% of cases, albeit after 3–5 years [4], and patients can develop pituitary insufficiency, brain vascular morbidity or secondary neoplasms. Stereotactic radiosurgery (RS) proved less effective results in macroadenomas, especially if they had already infiltrate the cavernous sinus. To obtain optimal efficacy, RS should thus be reserved to small well-defined lesions. The management of aggressive adenomas invading adjacent structures is a real challenge, as they rarely respond to any treatment.

In the presence of *ectopic secretion of ACTH*, surgical resection of the primary tumor is recommended. This results in the complete remission, especially in cases of benign tumor. Often, however, the tumor may already have metastasized, it may not be resectable, or it may not be identified despite extensive investigation (occult).

Bilateral adrenalectomy can be chosen as a final approach, reserved for patients who do not respond to surgical exploration of the hypophysis or radiation therapy, or when the source of ectopic ACTH is not found.

Adrenalectomy necessarily requires steroid replacement therapy for the rest of the patient's life, as with primary adrenocortical insufficiency. There is also a significant risk of developing Nelson's syndrome, which occurs in 5–10% of the patients, likely a subset with an aggressive phenotype, after adrenalectomy for Cushing's syndrome [4, 6]. It has been demonstrated that patients with invasive corticotrophinomas have a greater risk of subsequent (and earlier) development of Nelson's syndrome compared with less aggressive forms [7]. Prophylactic, conventional 3-field radiotherapy can be used to reduce the incidence of subsequent Nelson's and it should always be considered in the management of these patients [8]. When these approaches cannot be applied, a treatment is needed that has fewer side effects and can quickly reduce symptoms, and severe complications of hypercortisolism, aiming for the normalization of ACTH and serum cortisol values [9].

## 3. Medical Treatments

The therapeutic goal in the treatment of patients with ACTH-dependent Cushing's syndrome is normalization of plasma ACTH and serum cortisol values, tumor shrinkage and preservation of anterior pituitary function, in cases of pituitary ACTH-secreting tumor. Medical treatment can improve the clinical condition of patients with severe hypercortisolism pending surgery, during acute diseases (infections, psychosis, etc.), or in patients undergoing radiotherapy while awaiting the effects of the radiotherapy itself. In addition, patients with ectopic secretion of ACTH may be treated while expecting confirmation of the source, in the presence of metastatic cancer, or in patients who are not candidates for surgery for some reason.

Current drug-based therapy for CS includes drugs that act on the adrenal glands to reduce steroid synthesis, which therefore do not treat the underlying cause of the disease, and neuromodulators acting at the hypothalamic-pituitary level [10]. The existing treatments can be divided according to the site of action into adrenal acting drugs and in centrally acting drugs (Table 2).

### 3.1. Adrenal-Acting Drugs

Adrenal function must be carefully monitored, as excessive inhibition of steroidogenesis may cause adrenal insufficiency and may require the administration of small doses of glucocorticoids.

#### 3.1.1. Ketoconazole

This is the most currently used drug in patients with hypercortisolism. It is a synthetic antifungal drug that works principally by inhibiting the cytochrome P450 system and 17,20-lyase, which are involved in the synthesis and degradation of steroids. It has also been suggested that this drug may directly inhibit the pituitary corticotroph function, inhibiting ACTH secretion [11–13]. This is a fast-acting drug that quickly reduces urinary free cortisol (UFC) levels [14]. Its use has been reported as effective in 50% of patients with ectopic ACTH secretion. The most common side effects include gynecomastia, hypogonadism, gastrointestinal symptoms and reversible increases in liver enzymes. Severe liver toxicity is rare and liver function is usually restored after discontinuation. The drug does not inhibit the growth of the ACTH-secreting tumor.

#### 3.1.2. Metyrapone and LCI699

Metyrapone predominantly inhibits 11*β* hydroxylase and has been used either as a monotherapy, leading to a normalization of cortisol levels in 75–80% of patients, or in combination with other steroidogenesis inhibitors or with radiation therapy, achieving even higher efficacy [15, 16]. It is able to reduce cortisol production in patients with ectopic ACTH production and Cushing's disease. Side effects are dose-dependent, with the most common being hypertension, edema, increased acne and hirsutism in women due to its ability to inhibit the synthesis of aldosterone, resulting in an accumulation of its precursors with mineralocorticoid and weak androgen activity. However, when combined with ketoconazole, it offers a valuable and safe adjunct to control hypercortisolism. Recently, LCI699 [17], a novel orally active drug that inhibits at high doses the 11-beta hydroxylase activity (as well as aldosterone synthase) is under phase 2 evaluation for the management of hypercortisolism (http://clinicaltrial.gov/ identifier NCT01331239). 

#### 3.1.3. Aminoglutethimide

Aminoglutethimide is a potent reversible inhibitor of adrenal mineralocorticoid and glucocorticoid synthesis. It blocks cholesterol side-chain cleavage to pregnenolone, by inhibiting P450 enzymes. Side effects are skin rash, headache, a generalized pruritic rash, hypothyroidism, and goiter, and because of its toxicity is reserved for adrenal cancer. 

#### 3.1.4. Mitotane (o,p'-DDD)

It is a DDD (dichlorodiphenyldichloroethane) isomer and a derivative of DDT. A study of 177 patients showed a significant increase in the recurrence-free interval after radical surgery followed by mitotane when compared to surgery alone [18]. Mitotane blocks several steroidogenic enzymes, thus altering peripheral steroid metabolism, directly suppressing the adrenal cortex and altering cortisone metabolism. Its adrenolytic function appears at high doses (>4 g/day). It is effective in reducing UFC levels in 83% of treated patients [19, 20]. A 2006 study confirmed that most patients under mitotane treatment in a dose ranging from 4 to 6.5 g daily had dramatic increase in CBG levels, and serum cortisol levels can be elevated even when the circulating free cortisol level is not, thus making difficult to control its biochemical effect [21, 22]. It is commonly used in patients with adrenal carcinoma. Its main use is in patients with persistent disease despite surgical resection, those who are not candidates for surgery, and patients with metastatic disease.

Serum levels should be monitored to optimize therapy. The compound is distributed in the adipose tissue and has a long half-life. Gastrointestinal and neurologic symptoms are the most common side effects.

#### 3.1.5. Etomidate

Etomidate, an imidazole derivative, is an i.v. nonopioid anesthetic used for both induction and maintenance of anesthesia. It suppresses corticosteroid synthesis in the adrenal cortex by reversibly inhibiting 11-*β*-hydroxylase and 17,20 lyase at non-hypnotic doses. It has a very rapid onset of action and can be used in acute settings in patients with CS [23]. In addition, its intravenous administration makes it easily used in patients with no oral or enteral access. Studies and case reports support its use in patients with Cushing's syndrome. Chronic therapeutic use of ethyl-alcohol-containing Etomidate was effective for 8 weeks in a patient with ectopic CS and peritonitis [24]. In a 2001 case report, Etomidate was administered over 5.5 months, with daily dose modulation on the basis of serum cortisol levels. Suppression of steroidogenesis persisted for at least 14 days after cessation of the medication [25].

#### 3.1.6. Mifepristone (RU486)

Mifepristone is a synthetic steroid. It is a progesterone receptor antagonist and a powerful type-2 glucocorticoid receptor (GR) antagonist. It binds to human GR with an affinity three to four times higher than that of dexamethasone and about 18 times higher than that of cortisol. Its antiglucocorticoid effects are dose dependent. Mifepristone affects both the central actions of cortisol (negative feedback on CRH/ACTH secretion) and its peripheral actions and increases plasma ACTH and cortisol levels due to the loss of negative feedback of cortisol. This drug, currently used in the interruption of early pregnancy, was recently approved in patients with hyperglycemia induced by CS who are not candidates for surgery or where surgery has failed [26]. Medical literature suggests that mifepristone can improve clinical symptoms in 73–80% of patients [27] within one month after starting treatment. Castinetti et al. [28] reviewed the data of 37 treated CS patients (12 with EAS, 5 with Cushing's disease, the others affected by other causes of CS). A third of these developed hypokalemia. It was suggested that this resulted from cortisol stimulation of the mineralocorticoid receptor, while GRs were blocked by mifepristone. Spironolactone and potassium chloride replacement therapy can readily restore hypokalemia and blood pressure. Followup of efficacy and the onset of adrenal insufficiency (reported in 16% of 37 patients treated with Mifepristone) should only be clinical (weight, blood pressure, skin lesions) and biological (regular blood potassium sampling). The therapeutic dose adjustments should be based on these parameters. Mifepristone is often associated with the development of endometrial hyperplasia, so regular vaginal ultrasound is recommended in long-term treatment.

### 3.2. Centrally Acting Drugs 

In the last years several novel therapies have been studied with a view to the potential biochemical control and inhibition of pituitary tumor growth [29]. 

#### 3.2.1. Dopamine Agonists

Dopamine (DA) is a catecholamine hormone with a wide range of functions. DA receptors have been found in a variety of organs (pituitary, adrenals, brain, kidney, gastrointestinal tract, cardiovascular system), and possibly exert an inhibitory effect when activated. D2-receptor agonists inhibit pituitary hormone secretion, particularly PRL and proopiomelanocortin-derived hormones, and drugs such as cabergoline and bromocriptine effectively inhibit PRL secretion in prolactinomas. Studies on corticotroph adenomas have shown that 80% of these tumors express D2 receptors [30, 31]. In recent decades, published case reports and case series have demonstrated the effective use of DA agonists in persistent or recurrent Cushing's disease. 

The efficacy of bromocriptine in shrinking pituitary tumors was first reported in Nelson's syndrome and in the short-term treatment of CD [32–34]. However, the effect was not very strong, and response to long-term treatment was <30%. Cabergoline has a higher affinity for D2 receptors and a longer half-life compared to bromocriptine. In the short term [31] UFC levels normalized (40%) or decreased (20%) in a total cohort of 20 patients, 10 of whom underwent remission during long-term treatment (12–24 months) [35]. More recently a study demonstrated a 25% complete response to cabergoline in 12 patients with a followup of 6 months [36, 37] and confirmed that short-term treatment of CD with cabergoline improves cortisol secretion in half the cohort studied (30 patients), while long-term followup (37 months) demonstrated sustained effectiveness of cabergoline in 30% of subjects. 

There are a few documented cases of use of DA agonists in ectopic ACTH secretion. A study [38] describes 6 cases of ectopic tumors, three of which were not cured by surgery. UFC was normalized in two of these patients, although one exhibited treatment escape. A prospective study [39] evaluated the efficacy of cabergoline in monotherapy in patients with uncured CD, using sleeping midnight serum cortisol and the standard Low Dose Dexamethasone Suppression Test (LDDST) cut-off value as the response criteria. Cabergoline was effective and safe in 28% of 20 treated patients. This drug is generally well tolerated by most patients, and none of the subjects treated in these clinical trials showed signs of secondary heart dysfunction or valvulopathy, except a patient with a history of tricuspid regurgitation [40]. Cabergoline has also been described as having potential positive metabolic effects (pressure lowering, improvement of glucose tolerance), independently of its cortisol lowering effect. These findings renew interest in the potential use of dopamine agonists in Cushing's disease. 

#### 3.2.2. PPAR-*γ* Ligands

Peroxisome proliferative-activated receptor-*γ* (PPAR-*γ*), a member of the nuclear receptor superfamily, functions as a transcription factor mediating ligand-dependent transcriptional regulation [41]. PPAR-*γ* is expressed in several organs, and its administration is reported to inhibit tumor cell growth in the prostate and colon [42, 43]. Heaney et al. [41] documented the abundant expression of PPAR-*γ* in a series of ACTH-secreting tumor samples compared with aactivate PPAR-*γ* receptors, might be effective as a treatment for Cushing's disease. The literature evidence [44, 45] does not support this treatment, due to the lack of long-term benefit. Despite the finding of an initial reduction of ACTH and cortisol levels in a subset of patients with CD, clinical symptoms and biochemical parameters subsequently relapsed in this group of subjects. The administration of thiazolidinediones does not seem to be more effective than other currently available neuromodulators [45].

#### 3.2.3. Pasireotide (SOM230)

It is a somatostatin receptor (SSR) ligand with high binding affinity for multiple receptor isoforms (SST1-3 and SST5). SST5 and SST2 are highly expressed in ACTH pituitary adenomas, and animal studies documented that SSR mediates inhibition of cAMP and regulation of ACTH secretion [46]. A phase 2 trial [47] suggested that administration of Pasireotide for a 2-week period provoked a reduction in UFC in 76% of 29 patients affected by newly diagnosed, persistent or recurrent ACTH-dependent Cushing's disease. In a double blind, phase 3 study [48], 162 patients were randomly assigned to receive 600 mcg or 900 mcg subcutaneously twice daily. At 12 months, 26% and 15% of patients receiving, respectively, the higher and lower Pasireotide dose showed normalization of UFC levels. Serum and salivary cortisol and plasma ACTH decreased, and clinical features of hypercortisolism diminished. Side effects of this therapy included hyperglycemia (73%) and diabetes in 34% of patients, requiring treatment with glucose lowering medications in 45%. The other common symptoms were gastrointestinal disorders (diarrhea, abdominal pain, vomiting).

The significant results described in this 12-month phase 3 study support the use of Pasireotide as a targeted therapy for ACTH-secreting tumors. It is still not known if this treatment could act on pituitary tumor size. Octreotide, which acts predominantly on SSTR2 receptors, has not proven effective in inhibiting ACTH secretion in patients with Cushing's disease.

#### 3.2.4. Chemotherapy

In most cases, pituitary adenomas are benign slow-growing tumors. However, their rate of growth can be fast and they can be resistant to standard medical, surgical and radiation treatment [49], especially ACTH macroadenomas. The Crooke's cell variant of corticotroph adenoma has been described to be more aggressive and refractory to therapy, with a predisposition to malignant transformation [50–52]. When invasive tumors recur repeatedly despite radical surgery and postoperative radiotherapy, with widespread extrasellar extension, proximity to cranial nerves and critical blood vessels [2], combined cytotoxic therapy may be useful. It has also been suggested that early application of chemotherapy may be useful in patients who have already exhausted all surgical and radiotherapy options and are at high risk of malignant transformation [53, 54]. Kaiser et al. [55] reported a good response to cyclophosphamide, doxorubicin and 5-fluorouracil (5FU) in a patient with adrenocorticotroph tumor, with regression of the metastases. Kaltsas et al. [53] recommended the use of CCNU/5FU for relatively indolent tumor in the first instance. There have been partial and short-lasting responses to other combinations of chemotherapy agents [2], such as paclitaxel and etoposide in ectopic Cushing's syndrome [56]. In animal studies, cytotoxic hybrid compounds between the somatostatin analog vapreotide (no longer commercially available) and doxorubicin increased the effects of doxorubicin without increasing its toxicity [57].

#### 3.2.5. Temozolomide

Temozolomide (TMZ) is a second-generation alkylating cytostatic agent. Combined with radiotherapy, it is known to be effective in some patients with glioblastoma multiforme and cerebral metastases of malignant melanoma. It is administered orally, does not require hepatic metabolism for activation, and is able to cross the blood-brain barrier. TMZ promotes apoptosis of target cells and induces massive cell shrinkage and necrosis, depleting the DNA repair enzyme O6-methylguanine-DNA-methyl transferase (MGMT) in various cell types. Multiple studies suggest that reduced intratumor levels of MGMT predict responsiveness to TMZ. TMZ may also inhibit angiogenesis. Its use was firstly described in 2006 for the treatment of a pituitary carcinoma, and the first corticotroph adenoma was treated in 2007 [58]. Since then, more than 30 case reports on its use in ACTH-secreting pituitary tumors have been published, and on the whole described some type of positive response. Recently Raverot et al. [59] described four patients with ACTH tumors with 50% positive response after only four cycles, in terms of marked shrinkage of the pituitary tumor together with a markedly reduced extension of the vertebral metastases, and a drop in ACTH levels with clinical improvement. Curtò et al. [60] published a case report of a patient with a corticotroph carcinoma in whom a 90% reduction in the size of the tumor, and a stabilization of the metastases volume was documented after four cycles of TMZ. Dillard et al. [61] described a case of an aggressive 3 cm corticotroph adenoma refractory to multiple surgery and radiotherapy which showed a 60% regression in size after TMZ administration. TMZ treatment was generally well tolerated. It has been reported [59] that the initial response does not always correlate with long-term control of the disease and that the absence of MGMT expression may be associated with a better response. Tumor stabilization or reduction of tumor size can improve clinical outcomes, and it remains a last-line defense for life-threatening pituitary tumors.

#### 3.2.6. Retinoic Acid

Retinoids are a family of signaling molecules that are related to vitamin A (retinol) in terms of their chemical structure. The cell cycle is driven by complexes of cyclin-dependent kinases (CDKs) and cyclins. There is abundant evidence that retinoids, via various signaling pathways, inhibit cell-cycle progression in a variety of human cancer cells by directly or indirectly modulating cyclins, CDKs, and cell-cycle inhibitors. 

Retinoic acid (RA) has been studied in various types of tumor. Páez-Pereda et al. [62] examined its effects on human *in vitro* and mouse *in vivo *pituitary cells. RA inhibited ACTH biosynthesis only in tumorous corticotroph cells, while normal cells were unaffected. The authors concluded that RA inhibits ACTH synthesis by inhibiting POMC transcription through its activity on AP-1 and Nur77/Nurr1 and reduces the proliferation and survival of the corticotroph adenoma. It is thus of potential therapeutic use in CD [62, 63]. Castillo et al. [63] published an *in vivo* animal study in which retinoic acid or ketoconazole was administered to 42 dogs with Cushing's canine syndrome. A reduction in ACTH and alpha-MSH levels and pituitary adenoma volume was noted after 180 days of therapy with retinoic acid or ketoconazole, with similar results for both treatments. 

#### 3.2.7. mTOR Inhibitors

Mammalian target of rapamycin (mTOR) functions as a central element in a signaling pathway involved in the control of cell growth and proliferation. Everolimus is an mTOR inhibitor, and recent studies [64] have demonstrated its antineoplastic activity in several human cancers, mostly when associated with the long-acting repeatable (LAR) formulation of Octreotide in neuroendocrine tumors. Jouanneau et al. [65] hypothesized its use in pituitary aggressive adenomas and carcinomas. The authors described the effects of a combination therapy with everolimus (5 mg/day) and octreotide (30 mg/months) and studied mTOR expression in 1 pituitary carcinoma against 17 ACTH adenomas. Combined therapy did not control pituitary tumor growth or ACTH secretion, but the authors are waiting for more clinical cases before drawing any conclusions on this combined treatment.

#### 3.2.8. Tyrosine Kinase Inhibitors

Epidermal growth factor receptor (EGFR) activation, due to either mutation or ligand or receptor overexpression, is associated with a variety of human cancers. Approximately 60% of pituitary tumors, including ACTH-secreting adenomas, express EGFR. In pituitary corticotroph tumors expressing EGFR, p27^Kip1^, a cyclin-dependent kinase inhibitor, is down regulated. In a recent study [66], the authors hypothesized that the receptor could be a novel target for treatment of Cushing's disease, suppressing ACTH in corticotroph adenomas. In human ACTH-secreting tumors [67] gefitinib (a tyrosine kinase inhibitor targeting EGFR) was found to suppress *in vitro *POMC expression by approximately 95%. This effect was confirmed in canine corticotroph adenoma cells. Gefitinib effectively suppressed ACTH secretion and inhibited tumor growth in EGFR-expressing tumors *in vivo* (mouse model), in support of the *in vitro* results. 

#### 3.2.9. Combined Therapy

Since corticotroph adenomas express DA and SST receptors simultaneously, some authors hypothesized the use of DA agonists with SS analogues to reach a synergic effect in the treatment of ACTH-dependent CS. Recent studies [37] evaluated the association of cabergoline and ketoconazole, which normalized UFC in approximately 2/3 of patients not achieving a full response to cabergoline alone. In a prospective open-label, multicenter trial, Feelders et al. [68] administered Pasireotide in monotherapy followed by sequential addition of cabergoline and ketoconazole if UFC remained high after 28 and 60 days of treatment, respectively. At the end of the study 68% of the 17 treated patients showed complete biochemical control.

An innovative chimeric molecule that acts simultaneously on SST and DA receptors was created with a view to treatment of pituitary tumors. This compound (BIM-23A760) has been tested in a phase 1 and phase 2A study in 11 patients affected by acromegaly from GH-secreting pituitary adenoma, but the weak evidence of somatostatinergic activity led to the discontinuation of its development [69]. 

## 4. Conclusions

Management of persistent or recurrent CS is a challenge and medical therapy plays a critical role in the control of hypercortisolemia and associated symptoms. Unfortunately, this approach is not always successful. A multidisciplinary approach should thus be adopted, including chemotherapy, radiotherapy, neuromodulatory drugs, and hormone analogs to control tumor growth and associated symptoms. Ideally, management should be commenced in centers with appropriate experience and knowledge and involve a multidisciplinary team, including endocrinologists, neuroradiologists, dedicated neurosurgeons with expertise in pituitary tumor surgery (or general surgeons in cases of ectopic tumors), nuclear medicine physicians and oncologists.

Several studies have demonstrated that early application of medical treatment, possibly incorporating new therapeutic developments, may have improved effectiveness and led to more acceptable side effects. Nowadays, combination of tumor debulking, radiotherapy, medical treatment, and chemotherapy, appropriately and timely used, can avoid progression of an otherwise lethal condition. A better understanding of the pathogenesis of tumors underlying this puzzling syndrome is needed in order to help identify more effective and safe medical therapies. Control of hypercortisolemia should be obtained whenever possible, even in rapidly progressive disease, as small cell lung carcinomas, to reduce the associated complications (generalized infections, hypokalemia, diabetes, hypertension, psychotic reactions, and reduction of quality of life). Total bilateral adrenalectomy induces a rapid resolution of the clinical features. With low morbidity associated with laparoscopic adrenal surgery, this approach has been considered more frequently, and possibly even as main treatment in some individuals with Cushing's disease, especially when disease is severe or because of patient preference. Unfortunately the poor prognosis of this subgroup of EAS often makes the physician give up any drug control of the disease. We hope that the several offered noninvasive medical strategies can serve as a guide for the oncologists to improve the quality of life of their patients. 

## Figures and Tables

**Table 1:** 

Clinical features of hypercortisolism
Weight gain
Central obesity
Moon face
Purple stretch marks
Plethora
Easy bruising
Hirsutism
Acne
Severe fatigue and muscle weakness
High blood pressure
Depression
Cognitive impairment
Diabetes
Loss of libido
Menstrual disorders
Osteoporosis
Psychosis

**Table 2:** Medical treatments for Cushing's syndrome (in clinical use or investigational).

Drug	Mechanism of action	Dose (range)	Side effects	Safety monitoring
Ketoconazole	Inhibits steroidogenesis via inhibition of cytochrome P450 function	200–1800 mg per os (in divided doses, b.i.d.-t.i.d.)	Reversible liver dysfunction, severe liver toxicity, GI disorders, skin rash, loss of libido, impotence	Transaminase, testosterone, and SHBG in men
Metyrapone	Inhibits 11-*β* hydroxylase in the adrenal gland	750–6000 mg per os (in divided doses, t.i.d.-q.i.d.)	Hirsutism, acne, GI disorders, dizziness, hypertension, edema, hypokalemia	Androgens, mineralocorticoid, electrolytes
Aminoglutethimide	Prevents conversion of cholesterol to pregnenolone	250–750 mg per os (in divided doses, b.i.d.-t.i.d.)	Generalized, self-limiting itchy rash, nausea, dizziness, blurred vision, cholestasis, bone marrow suppression	Blood count, thyroid hormones, hepatic function, abdominal US
Mitotane	Inhibits steroidogenesis via inhibition of cytochrome P450; adrenolytic (high doses)	500 mg–12 g per os (daily)	Severe nausea, vomiting, diarrhea, rash, somnolence, ataxia, vertigo, dyslipidemia	Plasma mitotane, blood count, electrolytes, liver function, cholesterol
Etomidate	Inhibits 11-*β* hydroxylase and 17–20 lyase	<0.1 mg/kg/hr i.v.	Sedative effects, anesthesia	Monitoring by anesthesiologists
Mifepristone (RU-486)	Glucocorticoid, androgen, and progesterone receptor antagonist	300–1200 mg per os, daily dose	Hypoadrenalism, hypokalemia, hypertension, irregular menses, endometrial hyperplasia	Blood count, electrolytes, pelvic US
Cabergoline	D2 receptor agonist	1–7 mg per os, weekly dose	Nausea, vomiting, dizziness, valvulopathy	Echocardiogram
Octreotide	Somatostatin receptor agonist (isoform 2)	200–1000 mcg s.c. t.i.d., or LAR formulation 10–30 mg i.m. every 4 weeks	GI disorders, gallstones or biliary sludge, hyperglycemia, sinus bradycardia	Glycaemia, HbA1c, ECG, abdominal US
Pasireotide (SOM 230)	Somatostatin receptor agonist (isoforms 1, 2, 3, 5)	600–900 mcg s.c. b.i.d., LAR formulation under investigation	GI disorders, gallstones or biliary sludge, hyperglycemia or diabetes mellitus, sinus bradycardia	Glycaemia, HbA1c, Q-T interval, abdominal US
Retinoic acid	Inhibits POMC transcription and cell-cycle progression	No data *in vivo* in humans in Cushing's syndrome	Anaemia, mucocutaneous and ocular symptoms	Toxic effects of vitamin A, liver function, blood count
Rosiglitazone	PPAR-*γ* agonist	4–16 mg per os, daily doses	Weight increase, edema, somnolence, hirsutism	Blood count, transaminase, ECG, echocardiogram
Temozolomide	Alkylating agent	150–200 mg/m^2^ per os for 5 days once every 28 days, or 75 mg/m^2^ daily for 21 days with 7 day break	Bone marrow suppression, nausea, vomiting, dizziness diarrhea, rash	Blood count, liver and renal function, electrolytes
Gefitinib	Tyrosine kinase inhibitor	No data* in vivo *in humans in Cushing's disease	Fatigue, nausea, vomiting, stomatitis, bone pain, dyspnea, interstitial lung disease	Transaminase, pulmonary toxicity
Everolimus	mTOR inhibitor	5 mg/day	Bone marrow suppression, nausea, angioedema, GI disorders, extremity pain	Liver and renal function, blood count, glycaemia, HbA1c, lipid profile

b.i.d.: twice daily; t.i.d.: three times daily; q.i.d.: four times daily; i.v.: intravenous; i.m.: intramuscular; s.c.: subcutaneous; POMC: proopiomelanocortin; US: ultrasound; HbA1c: glycated hemoglobin; GI: gastrointestinal.
